# Supplementary material for: Asymmetric Reinforcement Learning Explains Human Choice Patterns in Decision-making Under Risk
Source: bioRxiv. 2026 Mar 11:2026.03.09.710615. Preprint. [Version 1] doi: 10.64898/2026.03.09.710615 (PMC13060910; doi:10.64898/2026.03.09.710615)
Supplement: Supplement 1 [file media-1.pdf]

Supplementary Information

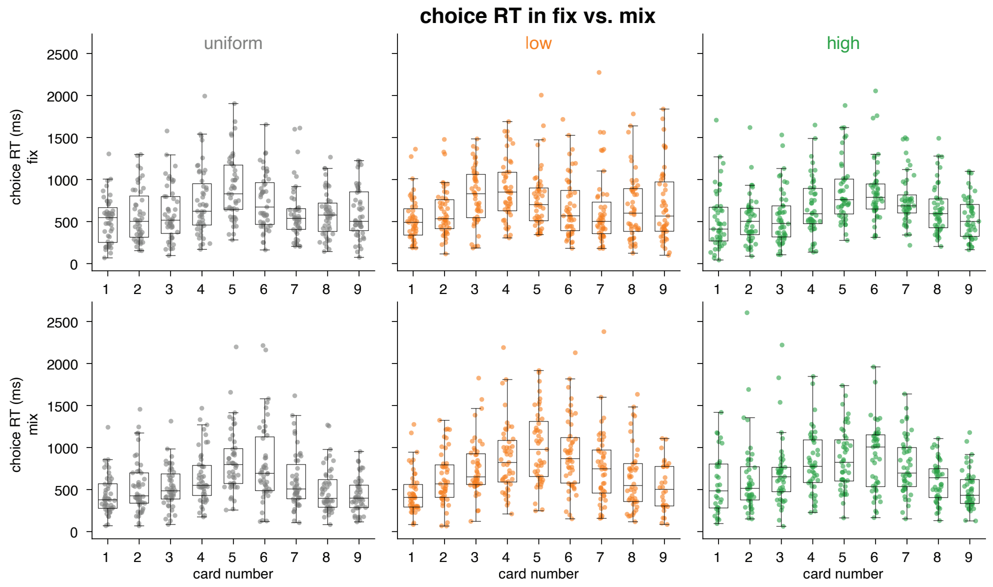

Fig. S1 Mean choice RT across participants and deck of cards for fix blocks (first row) and mix blocks (second row). Each marker indicates the mean choice RT of a single participant in each category.

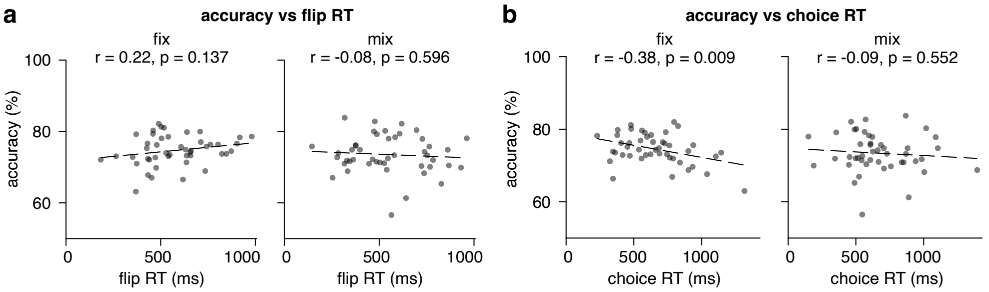

Fig. S2 Relationship between accuracy and the two RTs in fix and mix blocks. (**a**) Accuracy vs. flip RT in fix and mix blocks. Accuracy shows no reliable association with flip RT in either block. (**b**) Accuracy vs. choice RT in fix and mix blocks. In the fix block (the block with less uncertainty) accuracy decreases with longer choice RTs that shows the speed accuracy trade-off. Correlation coefficients (*r*) and *p*-values are shown in each panel.

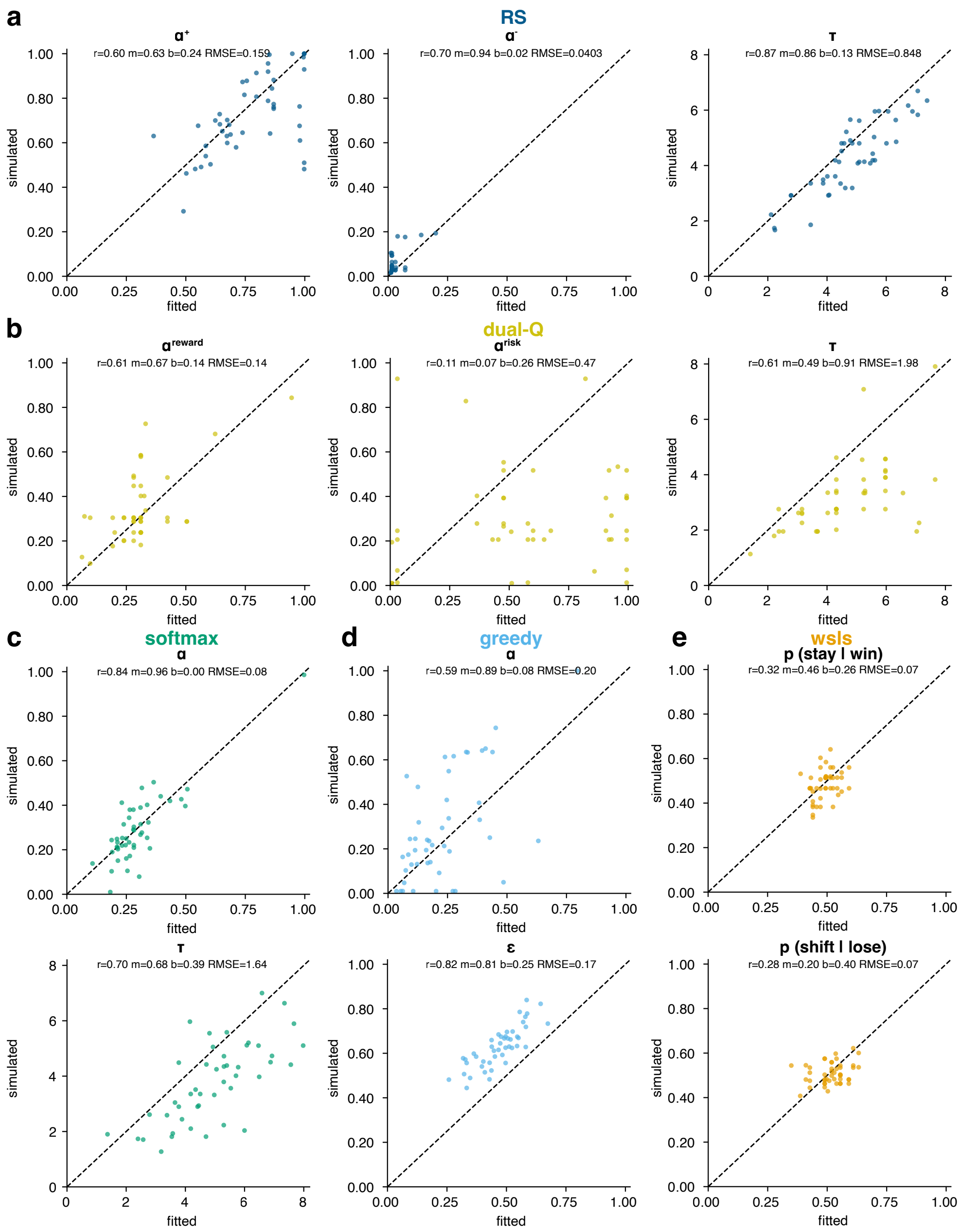

Fig. S3 Parameter recovery for models. Scatterplots show fitted vs. simulated values with recovery metrics (Pearson correlation r, slope m, intercept b, and Root Mean Squared Error). (a) RS parameters: α^+, α^-, τ. (b) Dual-Q parameters: α_{reward}, α_{risk}, τ. (c) Softmax parameters: α, τ. (d) Greedy parameters: α, ϵ. (e) WSLS parameters: $p(stay|win)$, $p(shift|lose)$.

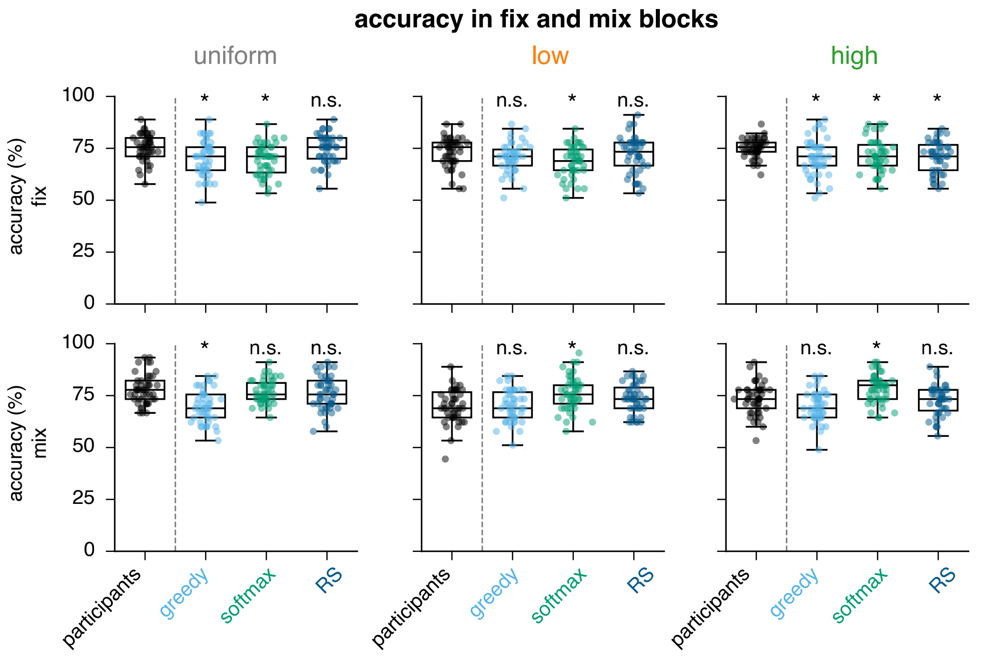

Fig. S4 Accuracy (%) for uniform, low, and high decks in fix (top row) and mix (bottom row) blocks for participants and each model. Participants vs. model comparisons are displayed above each model panel (* $p < 0.05$, n.s: not significant)

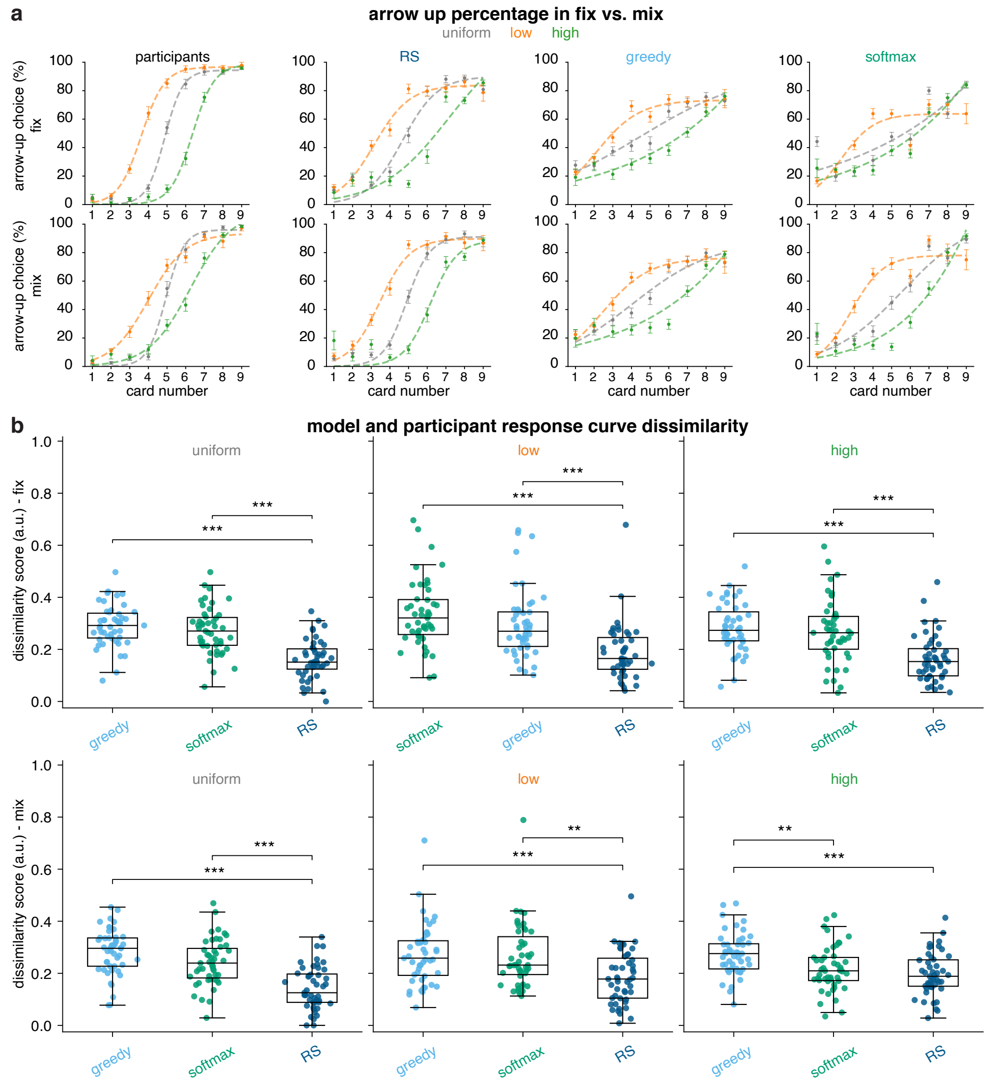

Fig. S5 (**a**) Arrow-up choice percentages across deck and card number in fix (top row) and mix (bottom row) blocks, comparing participant and model choices. (**b**) Response-curve dissimilarity scores between participants and models for each card decks (uniform, low, high) in fix (top row) and mix (bottom row) blocks (* $p < 0.05$, ** $p < 0.01$, *** $p < 0.001$).

Table S1 Risk for each *card*1 (participant's card) in the uniform deck.

$card1$	$N(card1)$	$45 - N(card1)$	$p(card2 < card1)$	$p(card2 > card1)$	Risk
1	5	40	0.000	1.000	0.000
2	5	40	0.125	0.875	0.125
3	5	40	0.250	0.750	0.250
4	5	40	0.375	0.625	0.375
5	5	40	0.500	0.500	0.500
6	5	40	0.625	0.375	0.375
7	5	40	0.750	0.250	0.250
8	5	40	0.875	0.125	0.125
9	5	40	1.000	0.000	0.000

Table S2 Risk for each *card*1 (participant's card) in the low deck.

$card1$	$N(card1)$	$45 - N(card1)$	$p(card2 < card1)$	$p(card2 > card1)$	Risk
1	9	36	0.000	1.000	0.000
2	8	37	0.243	0.757	0.243
3	7	38	0.447	0.553	0.447
4	6	39	0.615	0.385	0.385
5	5	40	0.750	0.250	0.250
6	4	41	0.854	0.146	0.146
7	3	42	0.929	0.071	0.071
8	2	43	0.977	0.023	0.023
9	1	44	1.000	0.000	0.000

Table S3 Risk for each *card*1 (participant's card) in the high deck.

$card1$	$N(card1)$	$45 - N(card1)$	$p(card2 < card1)$	$p(card2 > card1)$	Risk
1	1	44	0.000	1.000	0.000
2	2	43	0.023	0.977	0.023
3	3	42	0.071	0.929	0.071
4	4	41	0.146	0.854	0.146
5	5	40	0.250	0.750	0.250
6	6	39	0.385	0.615	0.385
7	7	38	0.447	0.553	0.447
8	8	37	0.243	0.757	0.243
9	9	36	0.000	1.000	0.000

Table S4 Linear regression results for choice across RS, Greedy, and Softmax models. Rows with $p < 0.05$ are in bold.

model	predictor	coef	std-err (clustered)	z	p
RS	baseline	0.294855	0.120822	2.4402	0.0880
	$\|\Delta Q\|$	**-0.091563**	**0.010892**	**-8.4026**	**<0.001**
	**deck - high**	**0.241338**	**0.037410**	**6.4512**	**<0.001**
	**deck - low**	**0.273379**	**0.037635**	**7.2639**	**<0.001**
	$\|5 - \textbf{card}\|$	**-0.255921**	**0.031071**	**-8.2376**	**<0.001**
	**log(trial)**	**-0.100699**	**0.031593**	**-3.1869**	**0.0010**
Greedy	baseline	0.260615	0.200887	1.2967	0.1956
	$\|\Delta Q\|$	**-0.036174**	**0.013870**	**-2.6084**	**0.0091**
	**deck - high**	**0.267142**	**0.041681**	**6.4093**	**<0.001**
	**deck - low**	**0.288583**	**0.041373**	**6.9724**	**<0.001**
	$\|5 - \textbf{card}\|$	**-0.262979**	**0.030742**	**-8.5540**	**<0.001**
	**log(trial)**	**-0.097196**	**0.030139**	**-3.2253**	**0.0012**
Softmax	baseline	0.264129	0.192614	1.3707	0.1699
	$\|\Delta Q\|$	**-0.040192**	**0.013890**	**-2.8930**	**0.0038**
	**deck - high**	**0.272669**	**0.041491**	**6.5723**	**<0.001**
	**deck - low**	**0.288234**	**0.041419**	**6.9575**	**<0.001**
	$\|5 - \textbf{card}\|$	**-0.262051**	**0.030838**	**-8.4978**	**<0.001**
	**log(trial)**	**-0.098116**	**0.030340**	**-3.2349**	**0.0012**

Table S5 Logistic regression results for choice across RS, Greedy, and Softmax models. Rows with $p < 0.05$ are in bold.

model	predictor	coef	std-err (clustered)	z	p
	baseline	-0.181	0.145	-1.246	0.211
	**ΔQ**	**5.765**	**0.773**	**7.454**	**<0.001**
RS	**deck - high**	**0.597**	**0.085**	**7.023**	**<0.001**
	**deck - low**	**-0.280**	**0.091**	**-3.087**	**0.002**
	log(trial)	0.033	0.034	0.970	0.333
	baseline	-0.113	0.110	-1.018	0.308
	**ΔQ**	**4.824**	**0.667**	**7.230**	**<0.001**
Greedy	**deck - high**	**0.343**	**0.082**	**4.179**	**<0.001**
	**deck - low**	**-0.285**	**0.083**	**-3.422**	**<0.001**
	log(trial)	0.014	0.028	0.506	0.612
	baseline	-0.066	0.097	-0.685	0.494
	**ΔQ**	**4.432**	**0.696**	**6.368**	**<0.001**
Softmax	deck - high	0.159	0.124	1.266	0.205
	**deck - low**	**-0.257**	**0.076**	**-3.374**	**<0.001**
	log(trial)	0.038	0.031	1.259	0.208

Table S6 Demographic characteristics of epileptic and healthy participants. Age is reported in categorical bins; gender reflects self-reported categories.

Category	Level	Epileptic (N = 10)	Healthy (N = 37)
Age	18–24	2	5
	25–34	4	27
	35–44	3	1
	45–54	1	2
	65+	0	2
Gender	Female	4	18
	Male	6	16
	Non-binary	0	2
	Prefer not to say	0	1
